# The Weight of Hyperion and PRISMA Hyperspectral Sensor Characteristics on Image Capability to Retrieve Urban Surface Materials in the City of Venice

**DOI:** 10.3390/s23010454

**Published:** 2023-01-01

**Authors:** Rosa Maria Cavalli

**Affiliations:** Research Institute for Geo-Hydrological Protection (IRPI), National Research Council (CNR), 06128 Perugia, Italy; rosa.maria.cavalli@irpi.cnr.it; Tel.: +39-075-501-422

**Keywords:** hyperspectral sensor characteristics, Hyperion image, PRISMA image, spectral unmixing, MESMA, spatial and spectral accuracy, urban surface materials

## Abstract

Following the success of the first hyperspectral sensor, the evaluation of hyperspectral image capability became a challenge in research, which mainly focused on improving image pre-processing and processing steps to minimize their errors, whereas in this study, the focus was on the weight of hyperspectral sensor characteristics on image capability in order to distinguish this effect from errors caused by image pre-processing and processing steps and improve our knowledge of errors. For these purposes, two satellite hyperspectral sensors with similar spatial and spectral characteristics (Hyperion and PRISMA) were compared with corresponding synthetic images, and the city of Venice was selected as the study area. After creating the synthetic images, the errors in the simulation of Hyperion and PRISMA images were evaluated (1.6 and 1.1%, respectively). The same spectral unmixing procedure was performed using real and synthetic images, and their accuracies were compared. The spectral accuracies in root mean square error were equal to 0.017 and 0.016, respectively. In addition, 72.3 and 77.4% of these values were related to sensor characteristics. The spatial accuracies in the mean absolute error were equal to 3.93 and 3.68, respectively. A total of 55.6 and 59.0% of these values were related to sensor characteristics, and 22.6 and 22.3% were related to co-localization and spatial resampling errors. The difference between the radiometric precision values of the sensors was 6.81 and 5.91% regarding the spectral and spatial accuracies of Hyperion image. In conclusion, the results of this study showed that the combined use of two or more real hyperspectral images with similar characteristics and their synthetic images quantifies the weight of hyperspectral sensor characteristics on their image capability and improves our knowledge regarding processing errors, and thus image capability.

## 1. Introduction

The first hyperspectral sensor was the Airborne Imaging Spectrometer (AIS), built in early 1980 [1]. It sampled the spectrum from 1.2 to 2.4 μm with 128 narrow (i.e., 9.3 nm wide) and continuous bands [1]. Three AIS surveys were carried out over the Cuprite Mining District (Nevada, USA) between August 1983 and 1984, and data analysis revealed the capability of hyperspectral data to identify and map surface minerals [2]. Goetz et al. [1] highlighted that more than 100 narrow and contiguous spectral bands acquired by a hyperspectral sensor could provide detailed information regarding every individual element in an image and increase the probability of finding a unique characteristic for any given element, which more effectively distinguishes it from the other elements in the image. This success initially led to the design and development of other airborne sensors (Table 1) and, later, satellite sensors [3]; the first satellite sensor was Hyperion, aboard the Earth Observation satellite platform (EO-1), which was launched in November 2000 by the U.S. National Aeronautics and Space Administration (NASA) [4] and decommissioned in March 2017 [3].

An updated list of satellite hyperspectral sensors includes 12 existing sensors; among them, 8 sample the spectrum from visible (VIS) to near infrared (NIR) [9]: the Compact High Resolution Imaging Spectrometer—CHRIS—aboard the PROBA platform was launched in October 2001 and funded by the European Space Agency [10]; the Atmospheric Infrared Sounder—AIRS—onboard the AQUA platform was launched in May 2002, and the overall project was managed by the JPL [11]; the Hyperspectral Imaging Satellite—HySI—onboard the IMS-1 platform was launched in April 2008 and funded by the Indian Space Research Organization (ISRO) [12]; the Hyperspectral Imager—HSI—onboard the HJ-1A platform was launched in September 2008 and funded by the China Center for Resources Satellite Data and Application (CRESDA) [13]; the Hyperspectral Imager for the Coastal Ocean—HICO—onboard the ISS platform was launched in September 2009 and funded by the United States Office of Naval Research [14]; the Hyperspectral Imager—HSI—onboard the FLORA platform was launched in 2016 [9]; Orbital hyperspectral satellites—OHSs—onboard OVS-1A/B platforms were launched in June 2017 [15]; the DLR Earth Sensing Imaging Spectrometer—DESIS—onboard the ISS platform was launched in August 2018 and funded by the German Aerospace Center (DLR) [16].

The four remaining sensors sample the spectrum from VIS to short-wave infrared (SWIR), including the Hyperion sensor [9]: the Advanced Hyperspectral Imager—AHSI—onboard the GF-5 platform was launched in May 2018 and funded by the People’s Republic of China [17]; PRecursore IperSpettrale of the Application Mission—PRISMA—was launched in March 2019 and funded by the Italian Space Agency (ASI) [18]; the Hyperspectral Imager Suite—HISUI—onboard the ALOS3 platform was launched in December 2019 and developed by the Japanese Ministry of Economy, Trade, and Industry (METI) [19]; the Environmental Mapping and Analysis Program—EnMAP-HIS—was launched in April 2022 by the DLR [20]. It is interesting to note not only that most new satellite hyperspectral sensors sample the spectrum from VIS to SWIR, but also that most countries are investing in this technology. In addition to these satellite hyperspectral sensors in Earth’s orbit, six other sensors were launched outside of Earth’s orbit (i.e., Comet 67P, Venus, Mars, Vesta, Ceres, and Lunar) which sampled the spectrum from VIS to SWIR [3]. 

In summary, since 1980, the detection and evaluation of the capability of hyperspectral data has been a challenge in research. For this purpose, some authors created synthetic data by simulating the spectral characteristics of hyperspectral sensors to assess their capability of characterizing coastal waters [21,22,23], distinguishing minerals [24,25], and predicting soil organic carbon [26], whereas others simulated the spectral and spatial characteristics of hyperspectral sensors to map soil property [27] and evaluate their capability of distinguishing soil from vegetation [28] and urban surfaces [29]. For example, Cavalli [29] created 60 synthetic images with two different spectral ranges (i.e., from VIS to NIR and from VIS to SWIR), with five different spectral resolutions (i.e., FWHM equal to 3, 10, 30, 50, and 100 nm), and with six different spatial resolutions (i.e., 1,5, 10, 100, and 250 m) and highlighted that sensor capability mainly depends on spatial resolution, secondarily on spectral range and mixed pixel percentage, and lastly on spectral resolution. However, very few researchers have spatially and spectrally compared synthetic images with “real” images [24,28] because the creation of synthetic data requires an accurate reference map and thus detailed knowledge regarding ground truth [30,31].

Therefore, most authors have exploited “real” images, on the one hand, by developing and/or applying methods and, on the other hand, by comparing the results that were obtained from different images. With regard to the first approach, because spectral unmixing analysis is the most widely applied method to hyperspectral images (e.g., [32,33,34]), some authors proposed methods to solve the unmixing problem (e.g., pixel purity index [35], N-FINDR [36], interactive error analysis [37]), or to estimate the endmember fractional abundances (e.g., [38]), whereas other authors developed methods based on spatial analysis (e.g., Spectral Angle Mapping—SAM [39] and Spectral Information Divergence—SID [40]). With regard to the second approach, the results obtained from hyperspectral data were compared with those obtained from other hyperspectral data (e.g., Hyperion images were compared with CHRIS [41], Hyperspectral Satellite TianGong-1 [42], and PRISMA [43] hyperspectral data), from multispectral data (e.g., CASI and MIVIS hyperspectral images were compared with ATM multispectral data [44], and PRISMA hyperspectral images were compared with Sentinel-2A multispectral data [45]), and from other data (e.g., AHSI hyperspectral data were compared with the GlobalLand30 land cover data set [46]; MIVIS hyperspectral image was merged with DEM [47]). However, there are many sources of error as the capability evaluated from real image is due to both the characteristics of the sensor and each step of image pre-processing and processing (i.e., calibration [7,48]; atmospheric [49,50] and geometric [51] corrections; dimension reduction [30]; selected method [52]; etc.).

In this paper, the combined use of synthetic images and two or more real images with similar characteristics was proposed to evaluate hyperspectral image capability. This research is a continuation of a previous study which aimed to estimate the capability of most multispectral and hyperspectral remote sensors to retrieve urban surface materials in the city of Venice [29]. For this purpose, Cavalli [29] only employed synthetic images that were created from a non-validated reference map and therefore could not be compared with real data. In contrast, in the present study, work began not only from a validated high-spatial-resolution reference map [53,54], but also from two hyperspectral images with similar spatial and spectral characteristics (i.e., Hyperion and PRISMA). These images were compared with corresponding synthetic images, which were created from the validated map, in order to assess the weight of the hyperspectral sensor characteristics on the image capability; to distinguish this effect from errors due to image pre-processing, processing, and validation steps; and to improve our knowledge of these errors. For these purposes, after creating accurate synthetic images of the city of Venice, errors in the simulation of the real images were calculated; when the accuracy was good, the same processing procedure was applied to the real and synthetic images (Section 2). The comparison of their results allowed me to assess the errors due to the spatial and spectral characteristics of these sensors; to know the pre-processing, processing, and validation errors; and thus, to improve the capabilities of hyperspectral images (Section 3, Section 4 and Section 5).

## 2. Materials and Methods

### 2.1. Study Area

**Figure 1:**
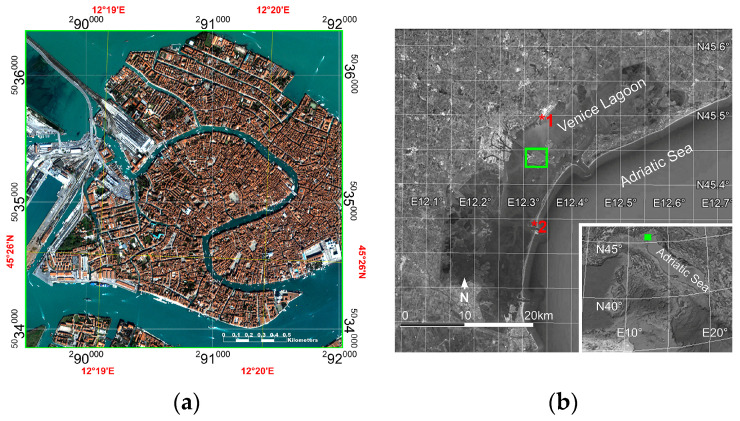
Study area: (**a**) IKONOS-2 image of study area; (**b**) study area location (green square) and locations of two intercalibration sites [57]: the runways of Marco Polo International Airport labeled with the number 1 and the Malamocco Golf Club labeled with the number 2.

The city of Venice was chosen as the study area because several field surveys, which were carried out to spatially and spectrally characterize urban surface materials, made it possible to create an accurate reference map and spectral library [55,56]. The city of Venice, which is famous worldwide, is located inside a large lagoon in the north-western Adriatic Sea which is named after the city (Figure 1b). The historical and artistic relevance of Venice can be traced back to the sea in terms of the significance of freight shipping in the city and, secondarily, the livelihoods that the sea provides. It was and remains a hub of intense commercial traffics. Venice is built on one hundred eighteen small islands and three hundred fifty-five bridges which connect them. A bridge also connects the mainland to the western part of the city, which is the only portion accessible by wheeled vehicles. In the rest of the city, people and objects move by foot or by boat. The buildings are close to one other and are separated by narrow streets, squares, streams, and canals (Figure 1a).

### 2.2. Real Hyperspectral Data of the City of Venice

Two satellite hyperspectral remote data with similar spatial and spectral characteristics were analyzed in this research: Hyperion data which were acquired on 7 June 2001 at 11:56 a.m. (GMT) in conjunction with a field campaign, and PRISMA data which were acquired on 7 July 2020 at 10:06 a.m. (GMT). Data regarding the study area were acquired using both sensors under clear-sky conditions. The characteristics of the hyperspectral sensors are summarized in Table 2.

**Figure 2:**
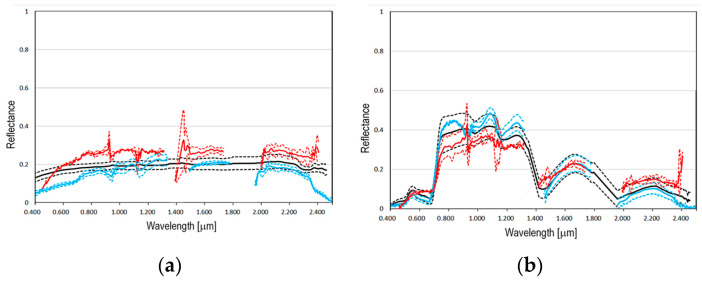
Comparison of the spectra acquired in situ (black) with the spectra of the Hyperion (red) and PRISMA (blue) atmospherically corrected images (the mean spectrum was plotted with a solid line, and the mean spectrum plus the standard deviation and minus the standard deviation were plotted with dashed lines): (**a**) asphalt spectra of the runways of Marco Polo International Airport (Figure 1b shows the site location using the number 1); (**b**) grass spectra of the Malamocco Golf Club (Figure 1b shows the site location using the number 2).

In terms of the Hyperion image, the data were provided as radiometrically corrected products. Therefore, after correcting the “SMILE” distortion effect [60], the image was atmospherically corrected by means of the FLAASH module [50], as implemented in the ENVI 5.6 software package, which incorporates the MODTRAN radiation transfer code [61]. With regard to the PRISMA image, the data were provided as atmospherically corrected products (Level2D [59]). Atmospheric correction data were validated using in situ spectral measurements acquired on two terrestrial intercalibration sites [57]: the runways of Marco Polo International Airport and the Malamocco Golf Club. Figure 1b shows the location of these sites which are, respectively, labeled with the numbers 1 and 2. The spectra of their surface materials (i.e., asphalt and grass, respectively) were compared with those derived from atmospherically corrected Hyperion and PRISMA images (Figure 2).

To co-localize the real images with the synthetic images and the reference map, two series of Ground Control Points (GCPs) were extracted from an IKONOS-2 panchromatic image resampled to 0.30 m which was utilized to create the reference map. Hyperion and PRISMA images yielded RMS errors of 0.118 and 0.109 pixels, respectively. Therefore, after applying the processing chain [62], the results were co-localized using these GCPs.

### 2.3. Synthetich Hyperspctral Data of the City of Venice 

Two synthetic images of the city of Venice were created, and their spectral and spatial characteristics matched the characteristics of the Hyperion and PRISMA hyperspectral images. In order to create these synthetic images, a reference map and spectral library were utilized.

**Figure 3:**
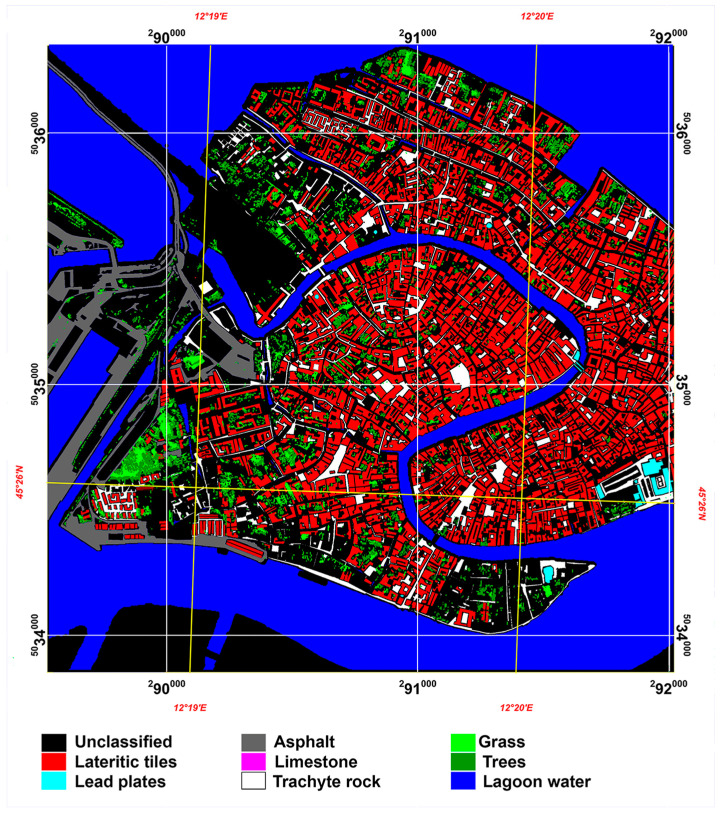
Reference map.

A previous map at the spatial resolution of 0.30 m was created by photo-interpreting an IKONOS-2 panchromatic image acquired on 2 April 2001 [56], and six surface materials were validated using ground truth data which were derived from different data [63]. Additional validation improved these previous reference maps, adding two surface materials to the six already mapped and removing all areas in which the presence of these materials was uncertain. The eight mapped surface materials were lateritic tiles, lead plates, asphalt, limestone, trachyte rock, grass, trees, and lagoon water (Figure 3). The reference map was divided into eight masks, and each mask included all of the pixels of the corresponding class.

**Figure 4:**
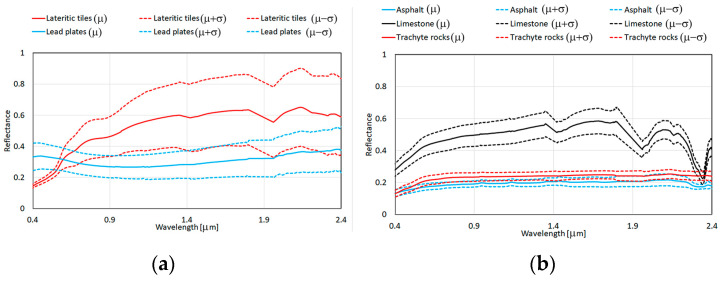

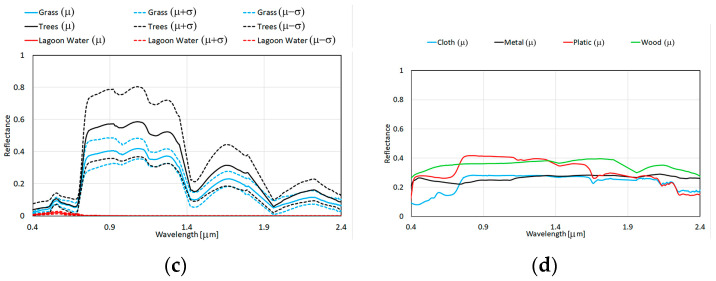
The spectral library (i.e., the mean spectra and the mean spectra plus and minus standard deviation values): (**a**) the spectra of lateritic tile and lead plate endmembers; (**b**) the spectra of asphalt, limestone, and trachyte rock endmembers; (**c**) the spectra of grass, tree, and lagoon water endmembers; (**d**) the spectra of cloth, metal, plastic, and wood endmembers.

The spectra of the surface materials of the city of Venice were acquired using a portable field spectrometer, Analytical Spectral Devices FieldSpec Full-Range Pro [55,64]. The characterization of surface materials was performed to sample their natural spectral variability at different city sites and to refine their spectral characterization in the laboratory [56] (Figure 4). Therefore, the spectral library, including the mean spectrum and its standard deviation of each material, was spectrally resampled according to the spectral characteristics of the Hyperion and PRISMA sensors.

The procedure proposed by [29] was exploited to create the synthetic images using 8 masks and the spectral library. To simulate the spectral variability of each endmember, the pixels of each mask were multiplied to the corresponding endmember spectra. Each mask was visualized as a chessboard with the same shape and size of the mask; its pixels that were associated with the black chessboard squares were multiplied by the mean spectrum plus the standard deviation, whereas those that were associated with the white chessboard squares were multiplied by the mean spectrum minus the standard deviation [29]. To simulate the variability in each mixed pixel, the remaining pixels were multiplied by two average spectra by utilizing the same procedure [29]. The first was the average spectrum of the 8 identified endmembers, and the second average was spectrum of some ancillary materials (i.e., wood, cloth, plastic, and metal; Figure 4d) plus the average spectrum of lagoon water. Therefore, the Hyperion and PRISMA synthetic images at spatial resolution of 0.30 cm were spatially resampled to 30 m.

To assess the spatial and spectral accuracy of the synthetic images, the spectral similarity between the reference spectra and the pixel spectra of the synthetic and real images was evaluated. As all supervised classifiers successfully employ different algorithms to measure similarity, the main classifiers (i.e., binary encoding, maximum likelihood, minimum distance, spectral angular mapper, and spectral information divergence) were taken into consideration to more effectively assess the spectral similarity [21]. Therefore, these classifiers were applied to real and synthetic images using the reference spectra as input; their results were sampled at the resolution of 0.30 m; the results obtained from the real images were geometrically warped using the selected set of GCP; their values were normalized, and the average values within each endmember mask were evaluated. The normalized average of the spectral similarity measurements, which was calculated for all of the pixels within endmember mask *k*, is expressed as

(1) $$aSSMn_{k}=\frac{\sum_{i=1}^{ni}\frac{\sum_{C=1}^{nc}\;\left(\frac{\left(SSM_{Ci}-\left(SSM_{Cmin\_k}\right)\right)}{\left(\left(SSM_{Cmax\_k}\right)-\left(SSM_{Cmin\_k}\right)\right)}\right)\;}{nc}}{ni}$$

where *nc* is the number of supervised classifications *C*, SSM*_Ci_* is the spectral similarity measurement which was calculated at pixel *i* within the endmember mask by the supervised classifier *C*, and SSM*_Cmin_k_* and SSM*_Cmax_k_* are the minimum and maximum values of the spectral similarity measurements which were calculated by the supervised classifier *C* within endmember mask *k*. The normalized average value of spectral similarity measurements was close to the value of 1 when the pixel spectrum of the synthetic and real images was spectrally similar to the reference spectrum, and when the pixel spectrum was not spectrally similar, it was close to the value of 0.

### 2.4. Processing Chain of Real and Synthetic Images

To retrieve urban surface materials from hyperspectral data, the most widely used spectral unmixing method is Multiple Endmember Spectral Mixture Analysis (MESMA) (e.g., [65,66,67]) because it searches for the best linear combination of endmembers for each mixed pixel, varying their number and types for each pixel, whereas linear spectral unmixing assumes that the set of endmembers present in a pixel is invariable. Therefore, it was applied to the synthetic and corrected real images by exploiting an open-source software application ‘VIPER2-tools’, which is an ENVI add-on [68]. Spectrum *S*′ measured at pixel *i* is given as a linear combination of endmember spectra:

(2) $${S^{\prime }}_{i}=\sum_{k=1}^{N}f_{ki}*S_{k}+\varepsilon_{i}$$

where *N* is the number of endmembers, *f_ki_* is the fractional abundance of the endmember *k* which was determined for the mixed pixel *i*, *S_k_* is the spectrum of the endmember *k*, and *ε* is the residual error which takes into account the spectral and spatial characteristics of the sensor, noise, and errors of the pre-processing and processing chains [30]. The fractional abundances are subject to two constraints: each fractional abundance must be positive $\left(f_{ki}\geq 0,\;k=1,\ldots ,\;N\right)$, and the sum of the abundances of each pixel must equal 1 $(\overset{N}{\underset{k=1}{\sum }}f_{ki}=1).$

The mean spectra of the 8 validated classes and the mean spectra of some ancillary materials (i.e., wood, cloth, plastic, and metal; Figure 4d) were exploited to determine the best linear combination of endmembers which was present in each mixed pixel of the real and synthetic images and to map their endmember fractional abundances. 

### 2.5. Spatial and Spectral Validation of the Results 

The most commonly used metric for the evaluation of the spectral accuracy of spectral unmixing results is the root mean square error (RMS) of the residual errors (*ε*) which is used to calculate the differences between the retrieved and reference endmembers (Equation (2), [68]). This metric was employed to spectrally validate the results that were obtained from the real and synthetic images. The average value of the residual errors of all of the pixels *i* within endmember mask *k* (RMS_k_) is given as

(3) $${\mathrm{RMS}}_{k}=\sqrt{\frac{\sum_{i=1}^{ni}{\left(\varepsilon_{i}\right)}^{2}}{ni}}$$

where *ni* is the number of pixels *i* within endmember mask *k*; RMS*_k_* values were calculated by applying “VIPER2-tools”, which determines the best set of endmembers by minimizing the RMS value for each pixel [68]. 

In order to spatially validate the fractional abundance maps retrieved from the real and synthetic images, the reference maps were transformed into reference fractional abundance maps following the procedure proposed by [29] and improved by [63]. The procedure is used to create reference fractional abundance maps by varying the spatial resolution of the reference maps and to evaluate their fractional abundance values in the entire range from 0 to 100% [63]. Each reference mask at a resolution of 0.30 m was sampled to a resolution of 30 m; these resampled masks only included pixels with an abundance of 100%; after returning these masks to a resolution of 0.30 m, the abundances of the remaining pixels within the initial mask would decrease linearly with increasing distance from a pixel with an abundance of 100%, and the distance between pixels with 100% and those with 0% would be equal to the spatial resolution of the sensor [63]. As the spatial resolution of the analyzed images was 30 m, the known abundance values obtained from the reference map at the spatial resolution of 0.30 m were equal to 100.

The difference between the reference fractional abundance maps and fractional abundance maps retrieved from the real and synthetic data was calculated with the mean absolute error (MAE), which is a common metric used [69]. The MAE for all of the pixels within endmember mask *k* was given as:

(4) $${\mathrm{MAE}}_{k}=\left(\frac{\sum_{i=1}^{ni}\left|A_{retrieved}-A_{reference}\right|}{ni}\right)$$

where the *A_retrieved_* and *A_reference_* are fractional abundances of the same pixels which were retrieved from remote and reference data, respectively. MAE_k_ was calculated in the range of abundances from 100 to 0% (MAE_k_-_Totals_), in the range from 100 to 50% (MAE_k-100–50%_), and in the range from 49 to 0% (MAE_k-49–0%_). This distinction of abundance range was performed as it divided pixels that were assigned to an endmember from those that were not assigned [70]. Before comparing fractional abundance maps retrieved from the real and synthetic images with the eight reference fractional abundance maps, MESNA results were resampled at 0.30 m, and those obtained from real images were geometrically warped using the selected series of GCP. 

**Figure 5:**
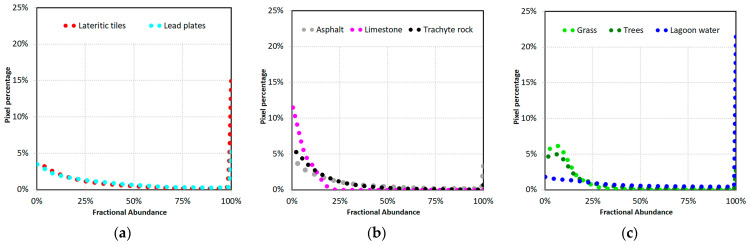
The histograms of fractional abundances obtained from the reference fractional abundance maps: (**a**) the histograms of lateritic tile and lead plate endmembers; (**b**) the histograms of asphalt, limestone, and trachyte rock endmembers; (**c**) the histograms of grass, tree, and lagoon water endmembers.

Some authors highlighted that co-localization and spatial resampling due to the comparison of different data at different spatial resolutions lead to errors in spatial validation [29,71]. In order to quantify these errors, the histograms of the reference fractional abundance maps and those resulting from spectral unmixing were compared [29], and their differences were evaluated using MAE values which were called H-MAE_k_. Therefore, the difference between the MAE_k_ and H-MAE_k_ values quantified the error in the co-localization and spatial resampling images. Figure 5 show the histograms of fractional abundances obtained from the reference fractional abundance maps.

## 3. Results

### 3.1. Accuracy of Synthetic Hyperspectral Images

**Figure 6:**
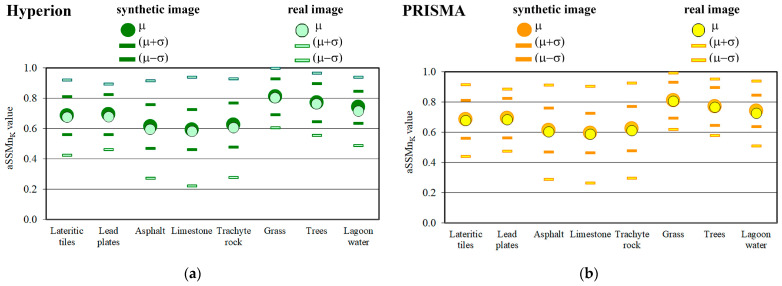
The comparison of the aSSMn_k_ values (i.e., mean values and mean values plus and minus standard deviation values) evaluated from real and synthetic images: (**a**) the values obtained from Hyperion images; (**b**) the values obtained from PRISMA images.

To evaluate the spectral and spatial accuracy of the synthetic images, spectral similarity measurements between the reference spectra and pixel spectra of the real and synthetic images were evaluated using five supervised classifiers, and the values were normalized; their mean values for each endmember were calculated within the corresponding mask (aSSMn_k_), which only included pixels in which the presence of the endmember was certain. In other words, spectral accuracy was determined by comparing the pixel spectra of the real and synthetic images with the reference spectra, whereas spatial accuracy was determined by only comparing the spectra of pixels within the reference masks. Figure 6 show the comparison of the aSSMn_k_ values (i.e., mean values and mean values plus and minus standard deviation values) obtained from the real and synthetic images.

Finally, to evaluate the accuracy of the synthetic images, the difference between the values of aSSMn_k_ obtained from the real and synthetic images (ΔaSSMn_k_) provided, on the one hand, the bias in the simulation of the real image (i.e., bias_sim_) and, on the other hand, the spatial and spectral accuracy of the synthetic image (i.e., 1 − bias_sim_). Table 3 shows the biases in the simulation of the real images. 

The biases in the simulation of the Hyperion image were slightly greater than those in the PRISMA image; the values obtained from the Hyperion image ranged from 2.7 to 1.0%, and the average value was equal to 1.6%, whereas the biases in the simulation of the PRISMA image ranged from 1.7 to 0.6%, and the average value was equal to 1.1%. Therefore, the spatial and spectral accuracies of the simulation of the real images were good: the average value obtained from the Hyperion synthetic image was equal to 98.4% and that of the PRISMA synthetic image was equal to 98.9%. As the sensors have similar spatial and spectral characteristics, this difference between the values obtained from the Hyperion and PRISMA images can be attributed to the radiometric precision of the sensors; it was shown that the radiometric precision of the Hyperion sensor is smaller than that of the PRISMA sensor (the sixth column in Table 2). 

In terms of the biases calculated from the Hyperion and PRISMA images, the grass and tree endmembers showed the smallest value, followed, in ascending order, by lateritic tiles, limestone, lead plates, trachyte rocks, asphalt, and, lastly, lagoon water endmembers.

### 3.2. Validation of Endmembers Retrieved from Real and Synthetic Images

**Figure 7:**
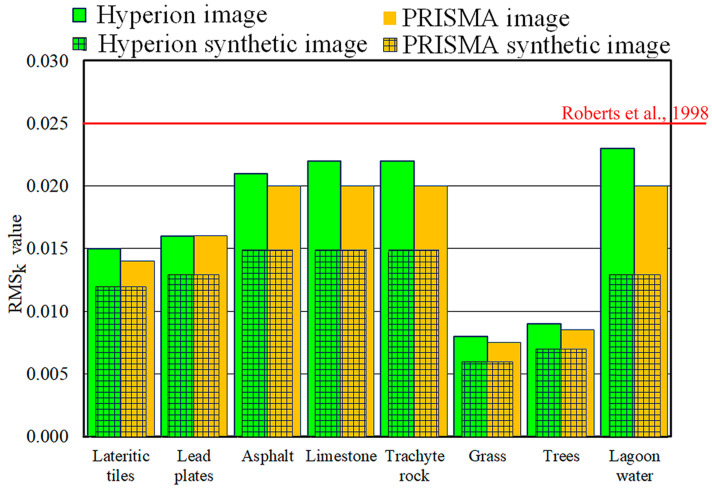
RMS*_k_* values obtained from real and synthetic (square pattern) images of Hyperion and PRISMA sensors [68].

As the spectral similarity measurements proved the good accuracy of the synthetic images, the same spectral unmixing procedure was applied to both the real and synthetic images, and their results (i.e., pixel constituent spectra and fractional abundance maps) were compared. As mentioned in the previous section, the pixel constituent spectra (i.e., endmembers) were spectrally validated by measuring the difference between the reference spectra and the spectrum of each image pixel, which was placed within the reference mask of the corresponding endmember. The spectral accuracies were obtained from the real and synthetic images using RMS_k_ values which were calculated by applying “VIPER2-tools” (Figure 7). 

The RMS*_k_* values obtained from Hyperion image were slightly greater than those obtained from the PRISMA image; the values obtained from the Hyperion image ranged from 0.023 to 0.008, and the average value was equal to 0.017, whereas the values obtained from the PRISMA image ranged from 0.020 to 0.008, and the average value was equal to 0.016 (Figure 7). As the focus of spectral unmixing is the determination of endmembers, its accuracy is attested by the RMS_k_ value [30], and Roberts et al. [68] proposed a threshold equal to 0.025. Therefore, the RMS_k_ values obtained demonstrate that the reference spectra used properly characterized all the endmembers present in the study area. 

The order of endmembers was the same for RMS_k_ values obtained from Hyperion and PRISMA images: the grass endmember had the smallest values, followed, in ascending order, by tree, lateritic tile, lead plate, asphalt, limestone, and trachyte rock endmembers; lastly, the lagoon water endmember had the highest values. As was mentioned previously, the choice of the reference endmembers was accurate for all of the endmembers, as all of the RMS_k_ values were below the threshold defined by [68]. However, the choice of the reference endmember of lagoon water was slightly less accurate than those of other materials. It is important to note that lagoon water showed the smallest reflectance values compared to all of the other endmembers (Figure 4); this means that its signal could easily be covered by noise [22]. 

Figure 7 also shows the RMS_k_ values obtained from the synthetic images (the columns with overlaid square pattern). The values obtained from the synthetic images were smaller than those obtained from real images; the values obtained from the Hyperion image were the same as those obtained from the PRISMA image; the order of the endmembers was the same as that highlighted for the real images, except for the position of the lagoon water endmember. In other words, the order of endmembers was due to the spatial and spectral characteristics of the sensors, except for the lagoon water endmember. This confirmed that the signal-to-noise ratio of the sensors (the sixth column in Table 1) weighed more heavily on the pixel spectra of the lagoon water endmember than on the other endmembers.

To determine the weight of the characteristics of the Hyperion and PRISMA sensors on the spectral accuracies, the ratio between the RMS_k_ values that were obtained from the real and synthetic images was evaluated. Table 4 shows the percentages of the RMS_k_ values, due to the sensor characteristics, added to the biases in the simulation of the real images. 

The percentage of RMS_k_ values caused by the Hyperion sensor characteristics were slightly smaller than those caused by the PRISMA sensor characteristics; the values obtained from the Hyperion image ranged from 81.3 ± 1.6 to 56.5 ± 2.2%, and the average value was equal to 72.3 ± 2.0%, whereas the values obtained from the PRISMA image ranged from 85.7 ± 1.9 to 65.0 ± 2.1%, and the average value was equal to 77.4 ± 1.9% (Table 4). 

### 3.3. Validation of Fractional Abundance Maps Retrieved from Real and Synthetic Images

**Figure 8:**
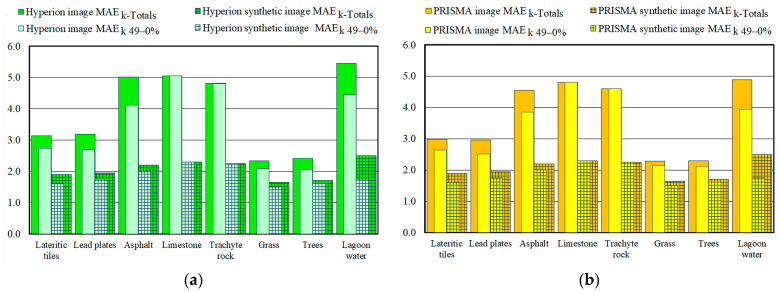
The comparison of the MAE_k_ values (MAE_k-49–0%_ values are superimposed on the MAE_kTotals_ values) evaluated from real and synthetic (square pattern) images: (**a**) the values obtained from Hyperion images; (**b**) the values obtained from PRISMA images.

The spatial accuracy of the fractional abundance maps was evaluated by measuring the differences between the fractional abundance retrieved from the real and synthetic images and the fractional abundance of the corresponding reference map using MAE_k-Totals_, MAE_k-100–50%,_ and MAE_k-49–0%_ values (Equation (4)). For each endmember, only the errors of the pixels placed within the corresponding reference mask were considered. Figure 8 show these values obtained from real and synthetic images of the Hyperion and PRISMA sensors.

As evidenced in the analysis of the spectral accuracies, the values obtained from the Hyperion image were slightly greater than those obtained from the PRISMA image; the values obtained from the Hyperion image ranged from 5.45 to 2.34, and the average value was equal to 3.93 (Figure 8a), whereas the values obtained from the PRISMA image ranged from 4.89 to 2.29, and the average value was equal to 3.68 (Figure 8b). Figure 8 also show the MAE_k_ values obtained from the synthetic images (the columns with overlaid square pattern). The MAE_k_ values obtained from synthetic images were smaller than those obtained from real images; the values obtained from the Hyperion image (Figure 8a) were the same as those obtained from the PRISMA image (Figure 8b). The difference between the values obtained from the real and synthetic Hyperion images ranged from 2.95 to 0.69, and their average value was equal to 1.87 (Figure 8a); the values obtained from the PRISMA images range from 2.51 to 0.59, and their average value was equal to 1.62 (Figure 8b). As mentioned above, the radiometric precision of the sensors could have caused the difference between the values obtained from the two real images.

As pixels with a fractional abundance greater than 50% made up a small percentage of all the endmembers (i.e., the percentages of pixels with fractional abundances greater than 50% that were quantified for the lateritic tile, lead plate, asphalt, limestone, trachyte rock, grass, tree, and lagoon water endmembers were equal to 28.85, 23.55, 5.93, 0.0, 12.26, 1.65, 8.97, and 45.80%, respectively), the MAE_k-100–50%_ values that were obtained from the real images were much smaller than the MAE_k-49–0%_ values.

To determine the weight of characteristics of the Hyperion and PRISMA sensors on the spatial accuracies, the ratio between the MAE_k-Totals_ values obtained from the real and synthetic images was evaluated (Table 5 and Table 6). 

As evidenced in the analysis of other values, the percentages of MAE_k-Totals_ values due to the spatial and spectral characteristics of the Hyperion sensor were slightly smaller than those from the PRISMA sensor; the values obtained from the Hyperion image ranged from 71.1 ± 1.7 to 43.8 ± 2.2%, and the average value was equal to 55.6 ± 2.0% (Table 4), whereas the values obtained from the PRISMA image ranged from 74.5 ± 1.5 to 47.8 ± 2.2%, and the average value was equal to 59.0 ± 1.9% (Table 5). The comparison of these values with the RMS_k_ values showed that sensor characteristics affected the spatial accuracy in smaller percentages than the spectral accuracies. The percentages of spatial accuracy which were not due to the characteristics of the Hyperion and PRISMA sensors were equal to 44.4 ± 2.0 and 41.0 ± 2.1% of the MAE_k-Totals_ values, respectively, whereas the percentages of spectral accuracy which were not due to the characteristics of the Hyperion and PRISMA sensors were equal to 27.7 ± 2.0 and 22.6 ± 1.9% of the RMS_k_ values, respectively. 

Regarding the percentages of MAE_k-Totals_ values which were due to sensor characteristics, the ranking of the percentage of MAE_k-Totals_ values was the same as those highlighted by the other values. The vegetation endmembers showed the highest values, followed, in descending order, by the building roofing material, street paving material, and lagoon water endmembers. As mentioned above, these smaller the percentages of MAE_k-Totals_ values due to sensor characteristics calculated for the lagoon water endmember were due to the small reflectance values of its spectra which could easily be covered by noise. Regarding the small the percentages of MAE_k-Totals_ values obtained from the street paving material endmembers, these values were due to the overestimation of their fractional abundances in the reference maps, as the road pavement pixels were mapped as being totally devoid of people and objects. 

**Figure 9:**
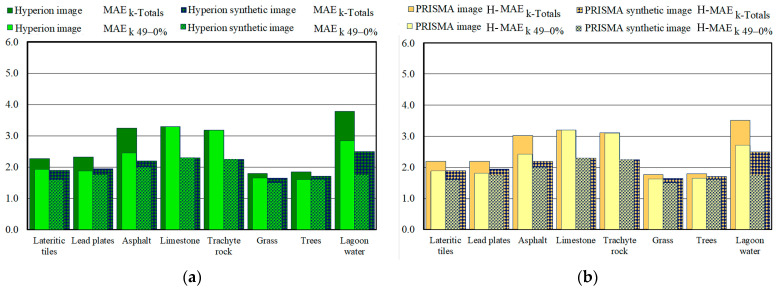
The comparison of the H-MAE_k_ values evaluated from real and synthetic (diamond pattern) images: (**a**) the values obtained from Hyperion images; (**b**) the values obtained from PRISMA images.

In the spatial validation, the comparison of different images at different spatial resolutions caused the errors in co-localization and spatial resampling. Therefore, the spatial accuracies of the fractional abundance maps were also evaluated without these errors. For this purpose, histograms of fractional abundance maps retrieved from the real and synthetic images were compared with histograms of the corresponding reference fractional abundance maps using H-MAE_k-Totals_, H-MAE_k-100–50%,_ and H-MAE_k-49–0%_ values (Equation (4)). Figure 9 show these values obtained from the real and synthetic images of the Hyperion and PRISMA sensors. 

Regarding the H-MAE_k-Totals_ values obtained using the histograms of fractional abundance maps, the values obtained from the Hyperion image were slightly greater than those obtained from the PRISMA image and ranged from 3.79 to 1.80, and the average value was equal to 2.72 (Figure 9a), whereas the values obtained from the PRISMA image ranged from 3.52 to 1.78, and the average value was equal to 2.61 (Figure 9b). The difference between the values obtained from the Hyperion and PRISMA images ranged from 0.27 to 0.02, and their average value was equal to 0.12 (Figure 9). Figure 9 also show the H-MAE_k_ values obtained from the synthetic images (the columns with diamond patterns). As evidenced by the analysis of the other values, the H-MAE_k_ values obtained from the synthetic images were smaller than those obtained from the real images; the values obtained from the Hyperion images (Figure 9a) were the same as those obtained from the PRISMA images (Figure 9b).

To determine the weight of characteristics of the Hyperion and PRISMA sensors on the spatial accuracies that were obtained using histograms of the fractional abundance maps, the ratio between the H-MAE_k-Totals_ values obtained from the real and synthetic images was evaluated. The percentages of these values caused by the Hyperion and PRISMA sensors were added to the error in the simulation of real data (Table 7).

Regarding the H-MAE_k-Totals_ values obtained using histograms of reference fractional abundance maps and fractional abundance maps, the percentages caused by the spatial and spectral characteristics of the Hyperion sensor were slightly greater than those caused by the PRISMA sensor characteristics and ranged from 93.0 ± 1.7 to 66.0 ± 2.2%, and the average value was equal to 78.2 ± 2.0%, whereas the percentages caused by the spatial and spectral characteristics of the PRISMA sensor ranged from 95.6 ± 1.5 to 71.0 ± 2.1%, and the average value was equal to 81.3 ± 1.9% (Table 7). Therefore, 21.8 ± 2.0 and 18.7 ± 2.1% of the MAE_k-Totals_ values were not due to the spatial and spectral characteristics of the Hyperion and PRISMA sensors, respectively. Regarding the percentages of the H-MAE_k-Totals_ values caused by sensor characteristics, the vegetation endmembers showed the highest values, followed, in descending order, by the building roofing material, street paving material, and lagoon water endmembers.

The errors in the co-localization and spatial resampling images were quantified by comparing the spatial accuracies caused by sensor characteristics which were obtained using the histograms of the fractional abundance maps (i.e., H-MAE_k_) and those that were obtained using the fractional abundance maps (i.e., MAE_k_). The errors in co-localization and spatial resampling retrieved from the Hyperion image were quite similar to those retrieved from the PRISMA image, and the values obtained from the Hyperion image ranged from 24.2 to 20.1%, and the average value was equal to 22.6% (the third columns of Table 5), whereas the errors in co-localization and spatial resampling data retrieved from the PRISMA image ranged from 24.3 to 19.9%, and the average value was equal to 22.3% (the third columns of Table 6). 

Regarding the errors in co-localization and spatial resampling, the vegetation endmembers showed the smallest values, followed, in descending order, by the building roofing material, street paving material, and lagoon water endmembers. It is important to note that the smallest errors in the co-localization and spatial resampling of the lagoon water endmember were due to its large, non-jagged shape and its surface which included 21.5% pure pixels and 44.9% mixed pixels which were characterized with fractional abundances of more than 50%.

## 4. Discussion

Despite the fact that research has been carried out for about 40 years in this field, there are many shortcomings yet to be overcome in all of the steps included in hyperspectral image pre-processing [49,72,73], processing [9,46,74], and validation [30,31,75]. However, not only the pre-processing, processing, and validation steps, but also sensor characteristics determine the capability of hyperspectral images. As these factors contribute to the image capability with different weights, it is very important to assess how much these factors weigh on spatial and spectral accuracy [30,31]. This evaluation is very important, because it provides the ranking of error values and quantifies errors that can or cannot be minimized. To evaluate the weight of some of these factors, in this paper, the comparisons of two hyperspectral images with similar spatial and spectral characteristics with their synthetic images were proposed. To overcome the lack of detailed knowledge regarding ground truth, this study began with the validation of reference maps. The presence of an endmember has to be certified in an area, and therefore, the map can only be used to attest to areas with certain land cover [54]. To map those areas with certain land cover, the spatial resolution of the reference map must be high [65,76]. Moreover, only these areas should be employed to spatially and spectrally validate the results [63].

In order to create a synthetic image which was spectrally and spatially comparable to the real one, the study area of Venice was chosen, because extensive knowledge of Venice provided an accurate spectral library and allowed the creation of a reference map at the resolution of 0.30 m, which was used to identify areas of certain attribution to eight endmembers. The Hyperion and PRISMA sensors were chosen as hyperspectral sensors with similar spatial and spectral characteristics. In order to spectrally and spatially validate the synthetic images, in this study, the exploitation of the procedure which was developed and employed to spectrally validate the data was proposed [21,22]. Therefore, the spectral validation of the synthetic images was carried out by taking advantage of the selected supervised classifiers which successfully employ different algorithms to measure similarity [21] and which, therefore, together offered a complete measure of spectral similarity between the reference spectra and the pixel spectra of the real and synthetic images. Meanwhile, the spatial validation was assessed by only comparing the spectra of pixels within the reference masks used to create the synthetic image. Once the good accuracy of the synthetic Hyperion and PRISMA images was demonstrated (i.e., the average errors in the simulation were equal to 1.6 and 1.1%, respectively), the same spectral unmixing procedure (i.e., MESMA) was applied to both the synthetic and real images. The average spectral accuracies of the results obtained from Hyperion and PRISMA images (i.e., the RMS_k_ values were equal to 0.017 and 0.016, respectively) were slightly greater than the values calculated by [63,68] (i.e., RMS values that were obtained from MIVIS and AVIRIS data were equal to 0.025 [63,68]). The analysis of spatial accuracies highlighted that the MAE_k-Totals_ values obtained from the Hyperion images were slightly greater than those obtained from the PRISMA image (i.e., 3.93 and 3.68, respectively). These values were smaller than those obtained from the same Hyperion image and other data using SAM: the MAE values that were obtained from ALI, ETM, Hyperion, and MIVIS data using SAM were equal to 17.3, 92.1, 26.7, and 10.7, respectively [56].

However, the most important results were obtained by comparing Hyperion and PRISMA images with their synthetic images; they are briefly summarized below:

- Sensor characteristics weighed 72.3 ± 2.0% and 77.4 ± 1.9% on spectral accuracy in the RMS_k_ values which were obtained from real Hyperion and PRISMA images, whereas the image pre-processing, processing, and validation steps weighed 27.7 ± 2.0 and 22.6 ± 1.9% on spectral accuracy in RMS_k_ values;
- Sensor characteristics weighed 55.6 ± 2.0% and 59.0 ± 1.9% on spatial accuracy in the MAE_k-Totals_ values which were obtained from real Hyperion and PRISMA images, whereas the image pre-processing, processing, and validation steps weighed 44.4 ± 2.0 and 41.0 ± 2.1% on spatial accuracy in the MAE_k-Totals_ values;
- The errors in the co-localization and spatial resampling of the images weighed 22.6 and 22.3% on spatial accuracy in the MAE_k-Totals_ values which were obtained from real Hyperion and PRISMA images;
- The difference between the radiometric precisions of the sensors weighed 6.81 and 5.91% on the RMS_k_ and MAE_k-Totals_ values which were obtained from real Hyperion image;
- The difference between the radiometric precisions of the sensors weighed 13.04 and 10.28% on RMS_k_ and MAE_k-Totals_ values which were obtained from real Hyperion image to determine the lagoon water endmember;
- The ranking list of endmembers of surface materials in the city of Venice according to their accuracies was determined by sensor characteristics, except for the endmember of lagoon water (i.e., vegetation endmembers showed the highest values of accuracy, followed, in descending order, by building roofing materials, street paving materials, and lagoon water endmembers).

As these results stimulate the continuation of the research, other areas of study that were acquired from more than two hyperspectral images are being explored.

## 5. Conclusions

Since 1980, the evaluation of the capabilities of hyperspectral images to identify and map surface materials has been successfully carried out using two different approaches: on the one hand, hyperspectral images have been compared with other hyperspectral images and/or multispectral images via the evaluation of their different capabilities; on the other hand, the spatial and spectral characteristics of hyperspectral sensors have been evaluated by creating synthetic data. However, the capability of hyperspectral images is determined not only by hyperspectral sensor characteristics, but also by all of the image pre-processing, processing, and validation steps. These factors contribute to the spatial and spectral accuracy of the results with different weights.

Therefore, in this paper, the combination of both approaches was proposed to evaluate the capabilities of Hyperion and PRISMA hyperspectral images to retrieve the urban surface materials in the city of Venice. The results demonstrate that the comparison of two or more hyperspectral images with similar characteristics with their synthetic images improves knowledge regarding hyperspectral image capacity through the assessment of how much sensor characteristics and the pre-processing, processing, and validation steps weigh on the spatial and spectral accuracy of the results.

## Figures and Tables

**Table 1:** The first airborne hyperspectral sensors.

The First Airborne Hyperspectral Sensor	Designed/Developed by (Country)	Year	Reference
Airborne Imaging Spectrometer (AIS)	Jet Propulsion Laboratory (JPL-USA)	1980	[1]
Airborne Visible Infrared Imaging Spectrometer (AVIRIS)	Jet Propulsion Laboratory (JPL-USA)	1984	[5]
Compact Airborne Spectrographic Imager (CASI)	ITRES (Canada)	1989	[6]
Multispectral Infrared Visible Imaging Spectrometer (MIVIS)	National Research Council (CNR-Italy)/Daedalus Inc. (USA)	1991	[7]
SWIR Full Spectrographic Imager (SFSI)	ITRES (Canada)	1992	[8]

**Table 2:** The characteristics of the Hyperion and PRISMA sensors.

Sensor	Spatial Resolution (m)	Bands	Spectral Region	Spectral Coverage (μm)	Radiometric Precision * (Signal/Noise)
Hyperion	30	220	VNIR	0.400–1.000	>144/1
			SWIR	0.900–2.500	>40/1
PRISMA	30	63	VNIR	0.400–0.972	>160/1 (>450/1 at 0.65 μm)
		171	SWIR	0.942–2.496	>100/1 (>360/1 at 1.55 μm)

* The values are as reported by Folkman et al. [58] for Hyperion and by Loizzo et al. [59] for PRISMA.

**Table 3:** Biases in the simulation of the real images estimated with ΔaSSMn_k_ values.

	Hyperion Images	PRISMA Images
Endmembers	bias_sim_	bias_sim_
Lateritic tiles	1.2%	0.9%
Lead plates	1.5%	1.3%
Asphalt	2.0%	1.4%
Limestone	1.4%	1.0%
Trachyte rock	2.0%	1.4%
Grass	1.0%	0.6%
Trees	1.0%	0.6%
Lagoon water	2.7%	1.7%

**Table 4:** The percentage of RMS_k_ values due to the sensor characteristics added to the biases in the simulation of the real images.

Endmembers	Percentage of RMS_k_ Values Due to Hyperion Sensor Characteristics	Percentage of RMS_k_ Values Due to PRISMA Sensor Characteristics
Lateritic tiles	80.0 ± 2.0	85.7 ± 1.9
Lead plates	81.3 ± 1.6	81.3 ± 1.6
Asphalt	71.4 ± 2.2	75.0 ± 2.1
Limestone	68.2 ± 2.7	75.0 ± 2.4
Trachyte rock	68.2 ± 2.2	75.0 ± 2.2
Grass	75.0 ± 1.7	80.0 ± 1.6
Trees	77.8 ± 1.7	82.4 ± 1.5
Lagoon water	56.5 ± 2.2	65.0 ± 2.1

**Table 5:** The percentage of MAE_k-Totals_ values due to Hyperion sensor characteristics added to the error in the simulation of real data and the percentage of MAE_k-Totals_ values due to errors in co-localization and spatial resampling of the image.

	Percentage of MAE_k-Totals_ Values That Were Obtained from Hyperion Images Due to	
Endmembers	Sensor Characteristics	Errors in Co-Localization and Spatial Resampling
Lateritic tiles	61.5 ± 2.0	22.8
Lead plates	61.1 ± 1.6	22.6
Asphalt	43.8 ± 2.2	23.9
Limestone	45.5 ± 2.7	24.2
Trachyte rock	46.7 ± 2.2	23.9
Grass	70.5 ± 1.7	21.2
Trees	71.1 ± 1.7	21.9
Lagoon water	45.9 ± 2.2	20.1

**Table 6:** The percentage of MAE_k-Totals_ values due to the PRISMA sensor characteristics added to the error in the simulation of real data and the percentage of MAE_k-Totals_ values due to errors in co-localization and spatial resampling of the image.

	Percentage of MAE_k-Totals_ Values That Were Obtained from PRISMA Images Due to	
Endmembers	Sensor Characteristics	Errors in Co-Localization and Spatial Resampling
Lateritic tiles	63.5 ± 1.9	22.4
Lead plates	65.7 ± 1.6	22.6
Asphalt	48.4 ± 2.1	24.3
Limestone	47.8 ± 2.4	24.1
Trachyte rock	48.8 ± 2.2	23.3
Grass	72.1 ± 1.6	20.6
Trees	74.5 ± 1.5	21.1
Lagoon water	51.1 ± 2.1	19.9

**Table 7:** The percentage of H-MAE_k-Totals_ values due to the sensor characteristics added to the biases in the simulation of real data.

Endmembers	Percentage of H-MAE_k-Totals_ Values Due to Hyperion Image Characteristics	Percentage of H-MAE_k-Totals_ Values Due to PRISMA Images Characteristics
Lateritic tiles	83.3 ± 2.0	86.0 ± 1.9
Lead plates	83.7 ± 1.6	88.2 ± 1.6
Asphalt	67.7 ± 2.2	72.6 ± 2.1
Limestone	69.7 ± 2.7	71.9 ± 2.4
Trachyte rock	70.5 ± 2.2	72.1 ± 2.2
Grass	91.7 ± 1.7	92.7 ± 1.6
Trees	93.0 ± 1.7	95.6 ± 1.5
Lagoon water	66.0 ± 2.2	71.0 ± 2.1

## Data Availability

Not applicable.
